# Natural solution to antibiotic resistance: bacteriophages ‘The Living Drugs’

**DOI:** 10.1007/s11274-014-1655-7

**Published:** 2014-04-30

**Authors:** Sabah A. A. Jassim, Richard G. Limoges

**Affiliations:** Applied Bio Research Inc., 455 Pelissier St., Windsor, ON N9A 6Z9 Canada

**Keywords:** Antibiotics, Bacteriophage, Biocontrol, Phage reprogramming technology, Phage safety and efficacy, Therapy

## Abstract

Antibiotics have been a panacea in animal husbandry as well as in human therapy for decades. The huge amount of antibiotics used to induce the growth and protect the health of farm animals has lead to the evolution of bacteria that are resistant to the drug’s effects. Today, many researchers are working with bacteriophages (phages) as an alternative to antibiotics in the control of pathogens for human therapy as well as prevention, biocontrol, and therapy in animal agriculture. Phage therapy and biocontrol have yet to fulfill their promise or potential, largely due to several key obstacles to their performance. Several suggestions are shared in order to point a direction for overcoming common obstacles in applied phage technology. The key to successful use of phages in modern scientific, farm, food processing and clinical applications is to understand the common obstacles as well as best practices and to develop answers that work in harmony with nature.

## Introduction

Throughout much of the twentieth century, antibiotics have been a primary defense against bacterial disease. Unfortunately, inappropriate and excessive use of antibiotics in animal husbandry is threatening their efficacy.

This review of current issues and causality concerning antibiotic resistance, points out some opportunities and uses for bacteriophage treatment and biocontrol in farm-related applications where the majority of antibiotics at subtherapeutic levels have been used as well as in clinical settings and summarizes some of the stumbling blocks that have emerged during past experimentation. The published work to date on bacteriophage therapy is supplemented with some suggestions for future direction in the field. By following best practices for the use of bacteriophage in farming and processing applications as well as in a potential human therapy and rising from challenges already and yet to be encountered, modern science can avoid a repeat of the resistance phenomenon encountered with antibiotics. A recap of the challenges for using bacteriophages in these varied but related settings along with some potential solutions or best practices is provided to aid future research.

## Agricultural antibiotics

Antibiotics are not only used to treat human illness but have also been used in livestock and poultry for more than half a century to control and treat diseases and in sub-therapeutic doses in animal feed, to promote growth and improve production of animal products (Stokstad and Jukes 1949; Page and Gautier 2012). This has resulted in the development of antibiotic-resistant bacteria (Lu 2004); the consequences affect everybody in the world and access to effective treatment for bacterial infections is urgently needed (Laxminarayan et al. 2013). Findings from recent studies using whole genome sequencing have now confirmed animal-to-human transfers of resistance genes (Harrison et al. 2013).

In agricultural industries, anti-microbial treatment in terrestrial animals reared for food production is for enteric and respiratory disorders in young animals and mastitis in dairy cows (Page and Gautier 2012). Anti-infective drugs for livestock now represent one of the largest markets in the world (Page and Gautier 2012). In the U.S. for instance, around 8 billion animals, (7.5 billion chickens, 300 million turkeys, and 100 million cattle) are treated by as many as 10 different antibiotics annually or during their lives (Martin 2004; Page and Gautier 2012). Antibiotics have also been used for prevention via feed or water to animals (Page and Gautier 2012; Laxminarayan et al. 2013). Preventive use can be anything from targeted interventions for controlling the spread of a diagnosed disease in a defined group of animals, to routine treatment of all animals during specific periods of stress such as weaning, after transportation, when combining new animals with a herd or mixing animals from different sources (Laxminarayan et al. 2013). With some exceptions, the antimicrobial classes used in agricultural industries are the same as in human medicine. However, some newer types of antimicrobials, such as carbapenems, oxazolidinones, and glycylcyclines are not used for animals reared for food (Laxminarayan et al. 2013).

Today, subtherapeutic antibiotics are routinely fed to livestock, poultry and fish on industrial farms to promote faster growth and to compensate for the unsanitary conditions in which they are raised (Emanuele 2010). Tetracycline, penicillin, erythromycin, and other antimicrobials that are important in human clinic are used extensively, in the absence of disease, for subtherapeutic purposes in today’s livestock production (Mellon et al. 2001; Page and Gautier 2012). In the US, overall use of antimicrobials for subtherapeutic purposes in animals rose by about 50 % between 1985 and 2001 (Gerber et al. 2007), and approximately 80 % of all antibiotics used in the U.S. are fed to farm animals (US Congress 2011). This was primarily driven by increased use in the poultry industry, where subtherapeutic antibiotic use increased from 2 million to 10.5 million pounds (907,185 kg to 4,762,720 kg) between the 1980s and 2001 which amounted to a dramatic 307 % increase on a per-bird basis (Mellon et al. 2001). In addition, where authorised, antibiotics used for growth promotion can generally be purchased over the counter without veterinary involvement (Khachatourians 1998; Manna et al. 2006; Laxminarayan et al. 2013). These practices have been decreasing the effectiveness of antibiotics in treating common infections, which has quickened in recent years, and the arrival of untreatable strains of carbapenem-resistant *Enterobacteriaceae*, indicates that the world is marching at the dawn of a post-antibiotic era (CDC 2013).

Additionally, it is estimated that approximately 75 % of all antibiotics given to animals are not fully digested and eventually pass through the body and enter the environment (Chee-Sanford et al. 2009), where they can encounter new bacteria and create additional resistant strains (Horrigan et al. 2002). With huge quantities of manure routinely sprayed onto fields surrounding confined animal feeding operations, antibiotic resistant bacteria can leech into surface and ground water, contaminating drinking wells and endangering the health of people living nearby (Clemans et al. 2011). Bacteria can also be spread by animals, birds and insects that come in contact with animal waste (Graham et al. 2009; Page and Gautier 2012). A considerable amount of pressure is being exerted on the natural microbial environment, including beneficial bacteria, human and animal nutrition and immunity, by the antibiotics provided to humans, animals and plants, as well as the spraying of antibiotics on fruit trees, resulting in dangerous superbugs (Phillips et al. 2004; Yan and Polk 2004; O’Hara and Shanahan 2006; Buffie et al. 2011).

## Economic impacts of subtherapeutic use of antibiotics

In 1998, the National Academy of Sciences calculated that increased health care costs associated with antibiotic-resistant bacteria exceed $4 billion each year in the U.S. alone, a figure that reflects the price of pharmaceuticals and longer hospital stays, but does not account for lost workdays, lost productivity or human suffering (Knobler et al. 2003; Westwater et al. 2003; U.S. Congress 2011). In 2009, Cook County Hospital and the Alliance for Prudent Use of Antibiotics estimated that the total health care cost of antibiotic resistant infections in the US was between $16.6 and $26 billion annually (U.S. Congress 2011). The WHO, the American Medical Association and the American Public Health Association have urged a ban on GPAs, arguing that their use leads to increased antibiotic-resistant infections in humans (Graham et al. 2007). Along with expected decreases in health care costs that would stem from reducing the number of drug resistant infections, there is evidence to show that eliminating GPAs would be profitable for both farmers and the public as a whole (U.S. Congress 2011). However, according to a study of Graham et al. (2007), the increased selling price of chickens fed GPAs did not offset the increased cost of the feed, resulting in a higher overall cost to the farmer. The study found that the use of GPAs resulted in an average loss in value per chicken of $0.0093, or about 0.45 % of total cost (Graham et al. 2007).

Today, many animal farmers do not use GPA’s, in large part because they don’t have to compensate for unhealthy conditions associated with confined animal feeding operations (Graham et al. 2007). On these types of farms such as organic poultry farms, *Enterococcus faecalis* and *E. faecium* resistance to antibiotics has been found to be significantly lower than on conventional poultry farms (Sapkota et al. 2011). These organic animals are raised in clean environments with adequate space to reduce animal-stress and the likelihood of infections (Graham et al. 2007). These types of farms may use antibiotics to treat acute infections in sick animals (Graham et al. 2007). These results do suggest that completely removing antibiotics from animal agriculture could effectively reduce resistance. In contrast, commercial interests have argued that their removal will have a significant impact on the cost of animal production and is unlikely to affect the risk to humans from antibiotic-resistant infections (Casewell et al. 2003). The economic impact of judicious use or a ban of antibiotics use in animals is difficult to measure, partly because exact figures for employees and profits in feed additives are not available and partly due to this conflicting evidence.

## Government intervention

The precise effect of agricultural antibiotic use on resistance levels in the general population is not known, but the evidence points to a link (Ganguly et al. 2011). In 2003, an expert committee convened by the WHO, the United Nations Food and Agriculture Organization and the World Organization for Animal Health concluded there is clear evidence of adverse human health consequences due to resistant organisms resulting from non-human usage of antimicrobials (Ganguly et al. 2011). These consequences include infections that would not have otherwise occurred, increased frequency of treatment failures (in some cases death), and increased severity of infections (FAO/OIE/WHO 2003). Today, governments around the world are taking action to address this issue. The Director-General of the WHO, said in 2011 that ‘The world is on the brink of losing these miracle cures (antibiotics). In the absence of urgent corrective and protective actions, the world is heading towards a post-antibiotic era, in which many common infections will no longer have a cure’ (Liljeqvist et al. 2012).

In Europe, restricted authorisation of antimicrobial types began several decades ago and in 2006 all growth promoting use was abandoned (Laxminarayan et al. 2013). In the US, the FDA has released draft guidelines on judicious use of antimicrobials in the rearing of animals for food production. These recommendations aim to reduce the overall use of medically important anti microbials and include veterinary oversight and consultation. If this guidance is adhered to, a gradual phasing out of growth promoting use is to be expected (Laxminarayan et al. 2013).

## The need for an alternative to antibiotics

Each year in the United States, at least 2 million people become infected with bacteria that are resistant to antibiotics. At least 23,000 people die annually as a direct result of these infections, while many more die from other conditions that were complicated by an antibiotic-resistant infection (Frieden 2013). Since the 1980s in the US, newly approved antibiotics have steadily declined and despite the increased awareness and redoubled efforts, the current R&D pipeline remains largely dry (Hughes 2011). The underlying economic factors make antibiotic development unprofitable, (Nathan and Goldberg 2005), since it is not commercially viable to develop new drugs if there is a high probability of their becoming ineffective soon after introduction (Liljeqvist et al. 2012). One of the major drawbacks is the inability to discover completely new antibiotics; those discovered over the last few decades have now been modified to produce new generic forms (Jose 2010), which is a disincentive to spending money on R&D.

Antibiotics were used in poultry industries to reduce *Salmonella* levels at each step of the production in the farms. Yet *Salmonella* remains the major cause of food-borne diseases worldwide, with chickens known to be the main reservoir for this zoonotic pathogen (FSA 2011; Bardina et al. 2012). It is the second leading cause of bacterial foodborne illness in the US and the great majority of these infections are associated with the consumption of products such as poultry and eggs contaminated with *Salmonella* (Foley et al. 2008). *Salmonella* have evolved several virulence and antimicrobial resistance mechanisms that allow for continued challenges to our public health infrastructure (Foley and Lynne 2008).

The emergence of infectious disease caused by drug-resistant bacteria requires alternatives to conventional antibiotics (Barrow and Soothill 1997; Alisky et al. 1998; Carlton 1999; Sulakvelidze et al. 2001). The search for new drugs is becoming critical because of the growing concern over the failing antibiotic drug discovery pipeline. There is a great deal of interest to investigate alternatives and natural antimicrobial agents, which has also increased due to consumer awareness about the use of chemical preservatives in foodstuff and on food processing surfaces.

## Bacteriophages

Bacteriophages (phages) are described as viruses that infect bacteria. Application of phages has been investigated extensively, such as in the indicator of fecal contamination (Endley et al. 2003) and against antibiotic resistant bacteria (Yosef et al. 2014).

### Lytic phage

When a virulent phage infects a host bacterium, it replicates much faster than the host cell. The whole cycle can be completed in 30–40 min. The phage is a parasite that depends on the host for its propagation, which is influenced by a variety of factors such as temperature, nutrients, light and other environmental forces (Jassim and Limoges 2013). It subverts the host’s biological function and utilizes the host machinery for reproduction. The host cell undergoes lysis and dies, simultaneously liberating a large number of progeny phages, which are each then ready to start another cycle by infecting new neighbouring bacterial cells. This cycle is known a lytic ‘virulent’ cycle.

The lytic cycle or ‘virulent phages’ fit in the class of ‘natural antimicrobial controlling agents’ and are arguably the most abundant biological entities on the planet. These are being exploited in various areas of biotechnology, including rapid bacterial detection (Stewart et al. 1993; McIntyre et al. 1996; Stewart et al. 1998; Favrin et al. 2001, 2003; Jassim and Griffiths 2007; Rees and Loessner 2009; Jassim et al. 2011), food bioprocessing (Jassim et al. 2012) and removal of bacterial biofilm (Hibma et al. 1997; Jassim et al. 2012).

Phages are known to have some advantages associated with human therapy over the use of antibiotics (Sulakvelidze et al. 2001; Sulakvelidze and Kutter 2005; Loc-Carrillo and Abedon 2011). The inexorable rise in the incidence of antibiotic resistance in bacterial pathogens, coupled with the disappointingly low rate of emergence of new, clinically useful antibiotics, has refocused attention on the potential utility of phages for biocontrol and preventing or treating human and animal disease.

### Lysogenic phage

Other particles, called lysogenic phages, are ‘temperate’ or dormant phages which may take the form of a ‘prophage’ by integrating with the viral DNA in the host chromosome. They become a part of the host cell and replicate along with the host chromosome for many generations, coexisting as opposed to lysing the host cell (Jassim and Limoges 2013). This phenomenon is called ‘lysogeny’, which also provides immunity against infection by further phage particles of the same type, ensuring that there is only one copy of phage per bacterial cell. The unique characteristics of lysogenic or ‘temperate’ phages and their potential for exploitation have been demonstrated in a system that restores antibiotic efficiency by reversing pathogen resistance to antibiotics (Edgar et al. 2012). These phages are genetically engineered to reverse the pathogens’ drug resistance, thereby restoring their sensitivity to antibiotics. Unlike conventional phage therapy, the system does not rely on the phage’s ability to kill pathogens in the infected host, but instead, on its ability to deliver genetic constructs into the bacteria and thus render them sensitive to antibiotics prior to host infection. The transfer of the sensitizing cassette by the constructed phage will significantly enrich antibiotic-treatable pathogens on hospital surfaces. This may hold key advantages to revive the use of old generation antibiotics leading to the use of phage biotechnology synergistically with antibiotics.

The lysogenic phage or ‘prophage’ will drive the adaptive evolution of bacteria to achieve more powerful virulence factors inherited from previously infected bacteria via transduction, i.e., the transfer of genetic material to a bacterial cell via phage infection (Campbell 1988; Verheust et al. 2010). Lysogenic phages serve as a driving force in bacterial pathogenesis, acting not only in the evolution of bacterial pathogens through gene transfer, but also contributing directly to bacterial pathogenesis at the time of infection (Wagner and Waldor 2002; Verheust et al. 2010). The data of Vojtek et al. (2008) has indicated that horizontal transfer of lysogenic phages among group A *Streptococcus* can occur across the M-type barrier; these data also provide further support for the hypothesis that toxigenic conversion can occur via lysogeny between species. This mechanism specifically allows more efficient adaptation to changing host challenges, potentially leading to fitter and more virulent clones (Vojtek et al. 2008). Other authors concluded that this may represent a potentially serious hazard to humans, animals and plants (Saunders et al. 2001; Verheust et al. 2010).

In viruses, recombinational repair is most often studied as it is manifested in the phenomenon of phage MR, whereas MR is the process by which viral genomes containing inactivating genomic damage, interact within the infected cell to form a viable genome (Michod et al. 2008). MR was found in many phages that infect *E. coli* (T1, T2, T5, T6, and phiX174) and *Salmonella typhi* (Viphage) (Blanco and Devoret 1973; Michod et al. 2008). The genome damage expressed as a lysogenic prophage is an error prone in nature and can be reactivated (Bhattacharyya et al. 1991; Michod et al. 2008). The restoration of impaired biologic activity can be caused by chemical reaction, thermal application, genetic recombination, or helper elements (Duenas and Borrebaeck 1995; Jassim et al. 1995; Maloy and Youderian 1996; Rieder et al. 1996; Maloy and Gardner 1998; O’Sullivan et al. 1998; Gupt 2008; Michod et al. 2008; Jassim et al. 2010).

It is noteworthy that lateral gene transfer virulence factors can also be accomplished through the lysogenic phages, which harbour a multitude of prophages and each phage-encoded virulence or fitness factor makes an incremental contribution to the fitness of the lysogen (Brüssow et al. 2004; Verheust et al. 2010). This will lead eventually to the evolution of pathogenic bacteria (Verheust et al. 2010). However, the phage could become a clinically useful therapy tool through understanding how to control the phage-resistant bacteria (Mizoguchi et al. 2003; Fischer et al. 2004). Subsequent studies revealed that not all phages replicate similarly and that there are important differences in the replication cycles of lytic and lysogenic phages (Sulakvelidze et al. 2001; Jassim et al. 2010).

The emergence of phage-resistant mutants is undesirable and the study of Mizoguchi et al. (2003) employs a continuous culture to investigate sequential mutations of both phage PP01 and its host cells *E. coli* O157:H7. The phage PP01, previously shown to efficiently and specifically lyse *E. coli* O157:H7, showed that co-evolution occurred to the phage PP01 reducing the phage lytic activity, therefore they decided to extend their research to find other O157:H7-specific phages. They also concluded that only through understanding and controlling the emergence of phage-resistant bacteria could phage become a clinically useful tool. It seems that broad-range phage O157:H7-PP01 resistance by clonal heterogeneity represents a new class of bacteria–phage interactions (Fischer et al. 2004). Furthermore, *S. enteritidis* strains did not produce viable phages when grown on particular hosts, which behaved as complexes of phages (Sillankorva et al. 2010). The latter authors have concluded this is most likely because of the presence of inactive phage-related genomes (or their parts) in the bacterial strains which are capable of being reactivated or which can recombine with lytic phages. In fact, some of the failures of phage therapy were due to bacterial mutations leading to resistance to phage infection (Barrow and Soothill 1997; Alisky et al. 1998; Carlton 1999; Sulakvelidze et al. 2001).

## Phage in therapy/bio-control (prophylaxis) applications

There are numerous reviews describing both the potential for and caveats associated with the employment of phages to treat bacterial infections, especially in clinical settings (Goodridge and Abedon 2003). Phage therapy is like other methods of biological control with some comfort in the reduction of the use of chemical agents against pathogens (Fujiwara et al. 2011). The advantages associated with phage therapy relative particularly to chemical anti-bacterial agents were also reviewed. (Sulakvelidze and Kutter 2005; Loc-Carrillo and Abedon 2011). Phages can be bactericidal, they can increase in number responding to the incidence of pathogens over the course of treatment, tend to only minimally disrupt normal flora, are equally effective against antibiotic-resistant bacteria, are often easily discovered, seem to be capable of disrupting bacterial biofilms, and can have low inherent toxicities. The exploitation of phages as a realistic approach in the control of pathogens has attracted considerable interest in recent years (Sulakvelidze et al. 2001; Merril et al. 2003; Jassim et al. 2012), because of the emergence of antibiotic-resistant bacteria.

Phage therapeutic applications in various aspects of human therapy and nonclinical settings are reported (Sulakvelidze and Kutter 2005; Brüssow 2007; Górski et al. 2007, 2009; Harper and Kutter 2009; Kutter 2009; Kutter et al. 2010; Abedon et al. 2011; Loc-Carrillo and Abedon 2011). Phage treatment in human eyes, ears and nose via inhalation was used at the Eliava Institute in Tbilisi for decades (Kutter et al. 2010; Abedon et al. 2011). Recently, phages have been suggested to be included in a nebulizer to treat bacterial lung infections in cystic fibrosis patients (Golshahi et al. 2008) or to be sprayed as dried phages in respirable powders for the treatment of pulmonary infections (Matinkhoo et al. 2011). The first controlled clinical trial of a therapeutic phage preparation was conducted in 2009 and showed efficacy and safety in chronic otitis targeting antibiotic-resistant *Pseudomonas aeruginosa* (Wright et al. 2009). A year later, in an evaluation of a phage treatment for chronic otitis infection in dogs, the results show once more that administration of this topical phage mixture leads to lysis of *P. aeruginosa* in the ear without apparent toxicity and that it has potential to be a convenient and effective treatment for *P. aeruginosa* otitis (Hawkins et al. 2010).

In the area of animal biocontrol and agribusiness options, phages have shown a remarkable success (Smith et al. 1987; Biswas et al. 2002; Sulakvelidze and Barrow 2005; Hawkins et al. 2010). Phages have been extremely effective at treating a number of bacterial infections in controlled animal studies, especially as a biocontrol agent in the prevention of food-borne illnesses, due to its target specificity, rapid bacterial killing and ability to self-replicate (Smith et al. 1987; Biswas et al. 2002; Hawkins et al. 2010). Phages have the potential to treat bacterial infections afflicting animals and in particular to prevent fatal *Escherichia coli* respiratory infections in broiler chickens (Huff et al. 2002a, b, 2003a, b). Aerosol spraying and intramuscular injection have given the best results over using oral delivery of phages via direct administration or addition to drinking water and/or feed (Sillankorva et al. 2012). This is may be due to gastric *p*H levels preventing the proliferation of phages (Spits 2009). Virulent antigen-specific phages have been used in an attempt to control *E. coli* O157:H7 in batch culture (Kudva et al. 1999). Loc-Carrillo et al. (2005) and Wagenaar et al. (2005) reported that phage therapy (biocontrol) reduces *Campylobacter jejuni* colonization of broiler chickens. Several studies have also addressed the use of phages to decrease *Campylobacter* and *Salmonella* concentrations on poultry (Goode et al. 2003; Atterbury et al. 2007; Kittler et al. 2013).

Veterinary therapy/biocontrol applications require the appropriate administration targeting specific bacteria, with a strategy that includes a comprehensive methodology, detailing the phage-host interactions, dose optimization and accounting for all chemical and physical factors (Jassim and Limoges 2013). In general, a deep understanding of intrinsic phage properties is critical to designing therapeutic interventions. The reduction of foodborne pathogens requires a comprehensive phage control program at the farm, where the animals are born, hatched or raised, before shipment to processing plants. Potential pre-harvest sources of foodborne pathogen contamination include breeder herds and flocks, hatcheries, contaminated feed and water, along with environmental sources and vectors, such as litter, animal caretakers, and insects (Bailey 1993; Nayak et al. 2003).

## Regulatory approval of phage therapy

Classical phage treatments used since the 1920s in the Soviet era is being investigated as another potential strategy (Potera 2013). Since phages are part of both gastrointestinal and environmental ecosystems (Topley and Wilson 1990), bactericidal phages may provide a feasible natural, nontoxic approach for controlling several human pathogens (Alisky et al. 1998). The safety of phages was further assured by Duckworth and Gulig (2002) who stated that there has been no evidence that exposure to phage particles, even those normally associated with disease-causing bacteria, can actually result in the occurrence of human disease. Nevertheless, Borysowski and Górski (2008) examined the safety of phage therapy, especially in immunocompromised individuals. They discussed the possible negative interactions with the immune system and the relative safety of the therapy compared to its effectiveness since phage resistant bacteria and some phage preparations, especially lysates, have been found to exert immunostimulatory activity. This problem is of great importance in phage therapy since immune response-mediated antibacterial activity may be substantially suppressed in immunocompromised patients. In addition, another safety aspect might be taken into consideration, phages replicate at the site of infection or wherever the host bacteria are present, while phages are absent in sterile areas, thus ensuring an optimal self-adjusting dose of phages which is not found in other modes of non-biological antimicrobial agents (Katsunori 2003). These arguments have helped to pave the way for phage therapy/biocontrol to become a broadly relevant technology, including veterinary, agricultural, and food microbiology applications.

It was for the treatment or prevention of human infections that phage therapy first caught the world’s imagination and which today is the primary motivation of the field (Zhukov-Verezhnikov et al. 1978; Biswas et al. 2002; Merril et al. 2006; Hawkins et al. 2010; Kittler et al. 2013). Nevertheless, the regulatory requirements for these types of live drugs, phages are still challenging (Potera 2013) and their uses might not extend to life-threatening infections. The recent USFDA (2006) approval of Listeria-specific phage preparations for food additives has opened the door to new applications of these natural bacterial killers. It is known that phages only infect and lyse bacterial cells and are harmless to mammalians (USFDA 2006). This has eventually led to the development of a phage related product which received regulatory approval from the FDA in 2011, as a natural antimicrobial for use in agro-food industry as GRAS and by US-FSIS as safe for use in animals (Sillankorva et al. 2012; Klumpp and Loessner 2013). In general, although the safety of phages has been strongly suggested by human phage therapy, it should be noted that some phages, notably when in the form of lysogens (prophages), have been recognized as important contributors to bacterial virulence, or as vectors in horizontal gene transfer through transduction (Verheust et al. 2010) as discussed further below.

Current information regarding studies being conducted and/or ongoing trials with the primary purpose of experimental therapy to treat, with the aid of phages, patients with various infections can be obtained from www.clinicaltrial.gov; http://www.clinicaltrial.gov/ct2/results?term=phage+therapy&Search=Search; Parracho et al. (2012). The national and international regulatory compliance and regulations being employed can be obtained from Parracho et al. (2012).

## Phage experimental evolution

Replication inside host bacteria by a lytic phage is a complex process consisting of a cascade of events involving several structural and regulatory genes (Sulakvelidze et al. 2001). Some therapeutic phages have unique yet unidentified genes or mechanisms responsible for their ability to effectively lyse their target bacteria. For example, a group of authors from the EIBMV (Adamia et al. 1990) identified and cloned an anti-*Salmonella* phage gene responsible, at least in part, for the phage’s potent lethal activity against the *S.* *enterica* serovar *typhimurium* host strains. In another study (Andriashvili et al. 1986), a unique mechanism has been described for protecting phage DNA from the restriction-modification defences of *Staphylococcus aureus* host strain.

Phage gene expression has been studied by many researchers (Gupt 2008). Use of genetic virus design/breeding which is a genetic manipulation of the virus genome has been reported (Duenas and Borrebaeck 1995; Rieder et al. 1996; Barrow and Soothill 1997; Alisky et al. 1998; O’Sullivan et al. 1998). Expression of the *ant* gene, to determine the lysis-lysogeny decision of phages was also reported (Maloy and Youderian 1996; Maloy and Gardner 1998). This provides a positive selection for and against DNA-binding: repression of *ant* can be selected by requiring growth of lysogens, and mutants that cannot repress *ant* can be selected by requiring lytic growth of the phage. The use of genetically engineered nonlytic phage to specifically target and deliver DNA encoding bactericidal proteins to bacteria was reported (Hagens and Bläsi 2003; Westwater et al. 2003). The genetically engineered phage exerted a high killing efficiency while leaving the cells structurally intact. The use of recombinant viral particles in some instances might raise some biosafety concerns by bringing and potentially disseminating new genetic traits among bacterial populations (Verheust et al. 2010).

The identification of bacteriolytic peptides derived from phage could rekindle interest in phage as a source of a new generation of agents for combating multidrug resistant bacteria and offer a starting point for new therapeutic agents that could potentially circumvent such problems (Bernhardt et al. 2001a, b). It was reported that phages produce lysins which break bonds in the bacterial cell-wall peptidoglycan structure just before release of phage progeny and that the lysins enzymes have killed bacteria in vitro within 5 s (Nelson et al. 2001; Schuch et al. 2002). Further work with lysins enzymes may produce an effective bactericide and enhanced rapid diagnostic tools.

The efficiency of the in vivo ‘therapy’ use of lytic phages relies mainly on how robust, rapid and what specific action phages are able to exert before the immune system of the body being treated will reduce them below the level of effectiveness (Abedon et al. 2011). Therefore, it seems that the less robust, un-optimized, phages have less chance to succeed in abolishing in vivo bacterial infection than their robust, optimized counterparts. Moreover, it seems that the in vitro challenge of the attacking phages against host bacteria might be limited by the availability of highly efficient and specific phages for challenging each pathogen successfully.

## Modern phage technology: obstacles and indications

Although phage therapy has been practiced for several decades in some of the former Soviet Union countries and Poland, there are still many doubts as to its ability to replace antibiotics (Edgar et al. 2012). They are not yet “magic bullets” and they might not work in certain settings (Sulakvelidze 2011). The development of obligate lytic phages may provide one modality to kill only specific pathogens without harming beneficial flora. There are other issues to address, including the potential in vivo elimination of phages, phage-neutralizing antibodies and phage-resistant mutants (Sulakvelidze 2011).

Human infections caused by pathogens transmitted from fish or the aquatic environments are quite common and depend on the season, as well as patients’ contact with fish and the related environment (Novotny et al. 2004). It is well known that fish and seafood are a potential source of many foodborne pathogens for human beings (Novotny et al. 2004). The effects of phage-host interactions in a commercially important fish pathogen were studied (Laanto et al. 2012). They reported that *Flavobacterium columnare* has developed resistance to 3 lytic phages associated with a decline in the bacterial virulence. They have hypothesised that this is due to antagonistic co-evolution factors reducing the virulence of bacterial pathogens outside of a host due to the associated costs of defending against lytic phages. This study represents the first report that phage-based therapies can provide a disease management strategy for columnaris disease in aquaculture (Laanto et al. 2012).

The recently discovered, Sputnik virophage is a satellite virus that inhibits replication of its target phage and thus acts as a parasite of that virus in aquatic environments (Jassim and Limoges 2013). These virophages may also coexist as the natural predator of the phages that target foodborne pathogens, perhaps transmitted from their aquatic environments by fish and seafood. Virophages in aquatic environments hijack virus DNA in order to replicate and often deform phage/virus particles, making them less infective (Jassim and Limoges 2013). We have found no published report of their existence or survival outside of aquatic environments, but if confirmed, it may help to explain why, according to the US Center for Science in the Public Interest (CSPI 2008), fish and shellfish are more likely to cause foodborne-illness than any other category of food product. Even if these virophages exist only in aquatic environments, much of their work to hamper the effectiveness of phages is already accomplished prior to the food harvest. These foods are also considered a potential entry source of foodborne pathogens into the home (Scott 2003). It is worthwhile to investigate this postulation and potential association of virophages with aquatic foodborne pathogens in order to aid the understanding of phage ecology and bacterial evolution in greater clarity, assisting in the application of phages in therapy and biocontrol of bacterial infections.

The development of phage resistance by bacteria is an issue facing scientists investigating phage-bacteria interaction. Phage-mediated transduction of bacterial genes likely reflects an infrequent mistake in the assembly of the phage particle, rather than a bacterial adaptation (Michod et al. 2008). The mechanism that caused the spread of antibiotic resistance genes between bacteria occurs most often by the gene transfer process of plasmid mediated conjugation and sometimes by phage-mediated transduction (Michod et al. 2008).

Phage interactions and/or to allow irreversible phage binding to the *E. coli* O157 antigen was studied (Kudva et al. 1999). It was found that the movement of virions in the LPS layer before DNA injection may involve the release and rebinding of individual tail spikes rather than hydrolysis of the O-antigen (Baxa et al. 1996). This would suggest that effective infection might require normal LPS, thus, phage mutations seem to originate by alternation of LPS structure (Mizoguchi et al. 2003). The importance of LPS of the outer membrane in controlling the fate of phage attachment and the consequent phage infection of the host cell was reported (Mizoguchi et al. 2003). It was inferred that the modification of LPS of the outer membrane of host bacteria may play a key role in controlling the phage-host interaction and consequently control phage infection.

In general, phage host interactions are dependent on the binding of tail proteins to specific bacterial surface receptors (Pelczar et al. 1993). It seems that the development of a successful phage against target bacteria must address the emergence of mutant strains, the phage binding and infection of bacterium not being controlled by a single receptor, and the many factors which contribute to phage resistance including alteration or loss of receptors for the target cell envelope (Heller 1992; Barrow et al. 1998; Biswas et al. 2002; Mizoguchi et al. 2003; Jassim et al. 2010). Thus, the efficient use of phages to control bacterial infections may require isolation of mutant host-specific phages that can adsorb to hosts that make shorter O-side chains (Kudva et al. 1999). Practical application might be hampered by factors such as the lack of broad-host phages and heterogeneity (Kutter 2005). The ecology of both phages and bacteria were also not understood, resistance, failure to neutralize gastric *p*H prior to oral administration, inactivation of phages by host immune responses and environmental contamination issues are other obstacles (Kutter 2005). It was also suggested that changes of the bacterial hosts used for maintenance of phages must be avoided as these can drastically modify the parameters of the phage preparations, including host range and lytic activity (Sillankorva et al. 2010; Sulakvelidze 2011). The generally poor efficacy of commercial phage preparations led to widespread criticism and disagreement about the effectiveness of phages in treating disease (Atterbury 2009).

Another drawback is the survival and persistence of phages on different surfaces due to the impact of external forces on phage-host interactions in their surrounding environments (Jassim and Limoges 2013). Phage virility is affected by physical and chemical factors associated with the microscopic food matrix and with the conditions of application including environmental factors and the distinct properties of the phage itself (EFSA 2009). All these aspects must be investigated and well characterized before an effective biocontrol agent can be established and marketed (Bardina et al. 2012). The success of phage biocontrol to greatly reduce harmful bacteria entering the food chain at farm level requires the production of virulent phages that can survive in extreme environments and having a broad host range for the target genus, while lacking bacterial virulence genes.

## Phage safety and efficacy for therapy

### Phage bactericidal activity

Phage biocontrol is applying specific phages to selectively reduce or eliminate susceptible bacteria from selected environments, including human and animal bodies, artificial environments, such as farms, factories, offices, hospitals, or in laboratory (Kurtböke et al. 1992; Grandgirard et al. 2008). The ability of phages to recognize precisely their target hosts, rendered them as favourable antibacterial agents because broad-spectrum antibiotics kill target bacteria along with other beneficial bacteria present in the farm or in the organism body, namely, animal intestinal flora (Merril et al. 2003).

Bacterial resistance to phages will unquestionably develop, although according to some authors (Carlton 1999; Inal 2003; Tanji et al. 2005) the rate of developing resistance to phages is approximately 10-fold lower than that to antibiotics (Sulakvelidze et al. 2001). Furthermore, many earlier studies demonstrated that classical application of phages in bacterial therapy or biocontrol is attainable in theory but in practice were not so successful, due to the lack of full coverage of target bacteria and the rapid emergence of bacterial mutations leading to complete resistance against phage infection (Barrow and Soothill 1997; Alisky et al. 1998; Carlton 1999; Sulakvelidze et al. 2001; Goodridge and Abedon 2003). Therefore, phage therapy or phage biocontrol were unsuccessful and eventually led to replacement of phage therapy with antibiotic treatment (Barrow and Soothill 1997). Scientific methodologies could be developed to deal with antibiotic resistance in bacteria using bacteriophage, however viral proteins would also integrate into human and animal society with unknown effect. Viral based therapy could potentially lead to bacterial development of viral resistance. It would be wise to approach such methodologies with caution in order to avoid repeating mistakes that were made with the improper use of antibiotics. Other authors have refuted these assumptions and concluded that the rate of developing resistance against phages can be partially circumvented by using several phages in one preparation or cocktail (much like using two or more antibiotics simultaneously) (Sulakvelidze et al. 2001). More importantly, unlike using trial and error with antibiotics, when resistance against a given phage occurs, the specialists can rapidly select through testing (in a few days or weeks) a new phage that is effective against the phage-resistant bacteria (Sulakvelidze et al. 2001).

Therapeutic phages have some other advantages over antibiotics (Sulakvelidze et al. 2001; Sulakvelidze and Kutter 2005; Loc-Carrillo and Abedon 2011), and phages have been reported to be more effective than antibiotics in experimentally infected animals (Smith and Huggins 1982). Like bacteria but unlike antibiotics, phages mutate and therefore can also evolve to counter phage-resistant bacteria (Matsuzaki et al. 2005). Because phages attack bacteria by attaching to receptors on the bacterial cell surface, phage-resistant mutants (which lack these receptors) are often less pathogenic than phage-susceptible bacteria (Inal 2003; Santander and Robeson 2007; Capparelli et al. 2010; Friman et al. 2011; Laanto et al. 2012).

Despite the attractions of phage therapy, scientific and logistical challenges remain. Wild-type phage particles are rapidly eliminated by the body’s reticuloendothelial (mononuclear phagocyte) system, so in order to enhance phages’ circulatory time and improve the efficacy of treatment; long-circulating mutants (Merril et al. 1996; Keen 2012) must be selected. Wild-type virion and distribution concerns relating to the scalability of phage therapy have also been discussed (Lu and Koeris 2011). More broadly, for phage therapy to be useful in clinical settings, a patient’s specific etiological agent would need to be rapidly identified and matched to the relevant phage(s) in a comprehensive pre-existing database. Because this scenario is inconsistent with how antibiotics are traditionally employed (Bull et al. 2002), new and interdisciplinary thinking involving bioinformaticists, health care professionals, and phage researchers, among others, would be required to make phage therapy practicable on a large scale.

For oral therapies to be optimized, the phages must be shielded with a non-immunogenic polymer such as polyethylene glycol (Kim et al. 2008). On the other hand, the pharmacokinetics of self-replicating agents such as phages, differ from those of normal drugs (Robert et al. 2000; Brüssow 2005) which needs further investigation. Study of phage-bacterial-host cell interactions such as those carried-out by Cairns et al. (2009) to improve understanding of phages in vivo pharmacokinetics, including relevant inundation, proliferation thresholds, optimisation of formulations and long-term stability data is required before it can be widely used within a clinical setting (Abedon et al. 2011; Ryan et al. 2011; Parracho et al. 2012).

It is also unclear how effective phages would be in treating diseases caused by intracellular pathogens (e.g., *Salmonella* species), where bacteria multiply primarily inside body cells where they are inaccessible to phages. It is possible that phages will have only limited utility in treating infections caused by intracellular salmonella in children (Kiknadze et al. 1986). It was found that the most successful route of administration for the treatment of systemic infections was via the parenteral route. Oral delivery is mainly used to treat gastrointestinal infections. However, in some cases phages can also reach the systemic circulation. Local delivery (skin, ears, and teeth) has proved extremely successful in the treatment of topical infections, as has the inhalation of phages for the treatment of lung infections (Ryan et al. 2011).

In order to ultimately incorporate phage therapy into a larger antibacterial arsenal, a regulatory framework must exist that allows phages to be utilized to their maximum potential. Classical phage therapy is a form of personalized medicine because specific phages (usually multiple phages combined as a multivalent cocktail) are carefully selected to treat a patient’s specific bacterial infection. Success rates from these customized phages are five-to-six fold higher than that of standardized phage products (Zhukov-Verezhnikov et al. 1978), so the use of personalized phage cocktails has historically been crucial for effective treatment. This is most likely because of the presence of inactive phage-related genomes in the host strains which are capable of being reactivated or which can recombine with lytic phages (Sillankorva et al. 2010).

### Phage pharmacological study

Despite the large number of publications on phage therapy, there are very few reports in which the pharmacokinetics of therapeutic phage preparations is delineated (Payne et al. 2000; Robert et al. 2000; Payne and Jansen 2003; Levin and Bull 2004; Brüssow 2005; Górski et al. 2006; Gill 2008; Cairns et al. 2009; Abedon and Thomas-Abedon 2010; Gill 2010; Abedon et al. 2011; Parracho et al. 2012). The studies of Bogovazova et al. (1991) and Bogovazova et al. (1992) suggested that phages get into the bloodstream of laboratory animals (after a single oral dose) within 2–4 h and that they are found in the internal organs (liver, spleen, kidney, etc.) in approximately 10 h. Also, data concerning the persistence of administered phages indicate that phages can remain in the body for relatively prolonged periods of time, *i.e.*, up to several days (Babalova et al. 1968). In one study, the time needed for the phage to reduce, eliminate or cure the target bacteria in infected animals was defined as a reduction of *Salmonella* concentration in the chicken cecum, and obtained when the phage was administered one day before or just after bacterial infection and then again on different days post-infection (Bardina et al. 2012). In comparison, calves and piglets with diarrhea due to experimentally administered pathogenic *E. coli* were cured within 8 h following phage administration (Smith and Huggins 1983). Hence, elimination of the pathogenic *E. coli* at the pre-harvest stage could play a significant role in preventing its introduction into the food chain (Tauxe 1997). These results would suggest that due to the phage short-term effect; the application would be optimized according to the type of chronic infection with the length of time before slaughter that is required to control the particular infection for the animals.

Another noteworthy issue regarding pharmacokinetic study is that phage-neutralizing antibodies were reported (Geller et al. 1998). This could be one of the principal reasons phages had failed as a therapeutic, through their supposed inactivation by pre-existing antibodies (Carlton 1999). Phage immune response was also observed in a mice study (Sabah A A Jassim unpublished data). Rats or humans can develop effective immunity against all introduced phages (Merril et al. 2006). It seems the pharmacokinetic aspects of phage therapy pharmacology need considerable research in order to obtain rigorous pharmacological data concerning both lytic and lysogenic phages, including full-scale toxicological studies, before lytic phages can be used therapeutically in humans. Overall, Kutter et al. (2010) has concluded that to provide an overview of the potential of phage therapy as a means of treating or preventing human diseases, there is a need to explore the phage therapy state of the art as currently practiced by physicians in various pockets of phage therapy activity around the world.

## Future directions

Phage development and producing preparations as antidotes or as a biocontrol from farm to fork, requires an understanding of the obstacles associated with the use of ‘live drugs’ or phages.

### Challenges

There is renewed optimism for phages as possible new ‘live drugs’ with hope to overcome the multi drug resistant bacteria problem. Surprisingly, despite the approval to use phages in food and medical industries by several international agencies FDA, GRAS, US-FSIS (see Regulatory approval of phage therapy), phages have not gained widespread acceptance as compared to commercially proven pharmaceutical antimicrobial agents.

The following summary outlines the key issues in phage biocontrol and treatment that scientists have already encountered both in the literature as well as in the laboratories. These can help to frame a platform from which past mistakes with both phages and antibiotics can be avoided.

Summary of key obstacles to best practices with phage in modern applications

- Heterogeneity and ecology of both phages and bacteria were not understood.
- Need to select highly virulent phages against target bacteria in the patient.
- Single phage preparations used to treat mixtures of different bacteria.
- Recognition as personalized medicine using a multivalent cocktail carefully selected to treat a patient’s specific bacterial infection(s).
- Lack of standardized lytic phages that can target only their host cell without using genetic modification.
- Genetically modified phages changing the composition of colonizing bacterial flora in humans, risk of subsequent development of active infections.
- Lateral gene transfer virulence factors and antibiotic resistance.
- Restriction modification degradation of phage DNA upon infection.
- Resistance mutations in bacterial genes for adsorption, lysogeny and lysogenic conversion. Strict safety standards for human therapies not met.
- Toxigenic conversion via lysogeny between species allowing more efficient adaptation of host, potentially leading to fitter and more virulent clones.
- Failure to appropriately characterize or titre phage preparations.
- Changes in the bacterial cell envelope for example, use of antibiotics in animal production that can cause disruption of microbial cell wall synthesis.
- Effect of environmental factors which all contribute to the complexity and unpredictability of phage-host interactions in the field such as UV light, chemical disinfectants, nutrients, pollutants etc.
- The isolation and the cultivation of phages from natural sources are time consuming and problematic for producing large amounts of active inoculums.
- Failure to characterize phage preparation, *i.e.*, to determine the virulence to the target.
- Failure to neutralize gastric *p*H prior to oral administration.
- Immunogenicity antibodies developed against phage.
- Presence of endotoxins in phage preparation leading to toxic shock in the patient.
- Pharmacokinetics of self-replicating agents differs from those of normal drugs.
- In vivo susceptibility of bacterial pathogens to phages is poorly understood and future research on more phage-host cell interaction needed to define the requirements for successful phage treatments.
- Many phage experiments done in vitro models need to be extrapolated to in vivo growth.
- Phages can be reproduced from a commercially available phage preparation, a challenge to commercialization.
- Intellectual property rights are challenging for the use of phage therapy in modern medicine and these can also trigger ethical discussions.
- In the healthcare system phage therapy is still seen as a cost and a social program rather than an economic driver.
- Phage sectors need more time to develop entrepreneurs and innovation in their sector.

### Phage reprogramming

Although most phages do not represent a threat to human health (unless they are carrying virulence factors), the use of recombinant viral particles in some instances might raise some biosafety concerns by bringing and potentially disseminating new genetic traits among bacterial populations (Verheust et al. 2010). Jassim et al. (1995) and Jassim et al. (2010) have described novel non-genetically modified phage breeding and design technologies, respectively, for previously resistant bacterial strains. It is of particular importance to determine the host range of the phages that will be used within the complicated animal environments, for example the use of antibiotics in animal production can generate cell wall deficient or cell wall disrupted bacteria. The bacterial cell wall is the most important part of the bacterial structure for the phage attachment, required to initiate bacterial infection. Phage technology was previously developed for cell wall deficient bacteria using non-genetically bred phages by a Jassim research team (Hibma et al. 1997). On the other hand, some phages can infect a number of bacteria strains, while others are more specific and will only infect a particular sub-strain. The evolutionary survival of viruses is attributed to five realities (Jassim et al. 1995; Jassim and Naji 2003; Jassim 2005; Jassim et al. 2010; Jassim and Limoges 2013):

- Genetic variability,
- Variety in means of transmission,
- Efficient replication within host cells,
- Ability to remain dormant within the host (lysogeny),
- Environmental or external forces.

**Fig. 1:**
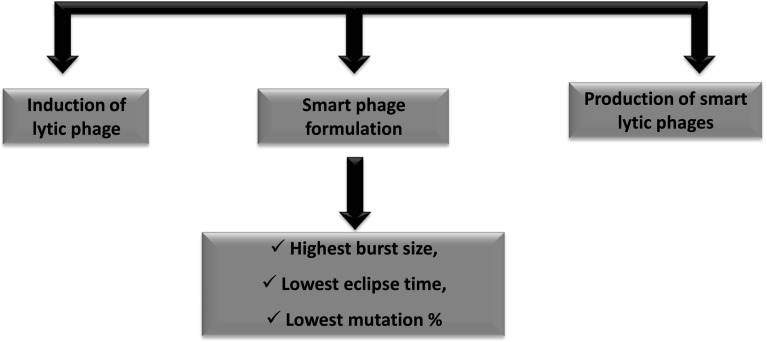
Non-genetic phage reprogramming technology to produce smart lytic phage (*Source*: Applied Bio Research Inc.)

Based on the above concepts, phage selectivity cultures (Jassim et al. 1995) and phage design technology (Jassim et al. 2010) were developed to address phage-host interactions and to produce highly lytic phages with no or far less phage-resistant mutants, along with broad host targeting capabilities. These methods do not employ genetic modification, to breed “re-tailored” wild phages on the host cells in order to gain newly bred sub-strains of phages which are able to overcome the host defence mechanisms in order to infect previously resistant bacteria and to play an important role in future applications (Jassim et al. 1995; Hibma et al. 1997). Newer methodologies are used to reprogram phages again without genetic modification, to possess auxiliary mechanisms for phage adherence/binding and uptake that are critical for plaque formation, in order to gain new sub-strains of phages able to infect parent resistant host cells. This non-genetic approach of the technology is environmentally-driven and so mimics natural selection or evolution of the phage by reproducing vast numbers of mixed populations of the most robust wild-type phages. Phage reprogramming technology was developed (Fig. 1) to permit a better selection and adaptation of robust lytic phages for each potential application. This technology is capable of converting naturally occurring wild phages to smart phages with a broader range of host specificity that can overcome a bacterium’s resistive defense mechanisms and completely destroy the target bacterial cell. These findings encourage new optimism and a re-evaluation of the potential for phage therapy.

## Discussion

Phages are naturally occurring predators of bacteria. They can be effective antibacterial agents due to their specificity against a particular bacterial species and lack of impact on other microflora. However, the potential problem still exists, that just as bacteria are able to become resistant to antibiotics, they may also be able to develop resistance to phages. Thus during the course of phage treatment, the etiologic agents should be continuously monitored for phage susceptibility and if phage resistance is developed, the subject phages can be replaced with different phages, lytic against the newly emerged, phage-resistant bacteria mutants.

The lysogenic phage contributes by providing axes force in bacterial pathogenesis and contributing in the bacterial pathogens evolution through horizontal gene transfer. Therefore, the development of a successful phage therapeutic against medically important human and animal pathogens must address the emergence of all of these mutant strains. Specialists must focus on modelling phage systems in natural ecosystems to prevent bacterial resistance to the phage, which must be addressed before using phage therapy/biocontrol.

Furthermore, no consensus exists on how quickly phages should produce results to identify patients who really need phage therapy. Should the research need to invest in developing smart phages that can produce results to prevent phage resistance to the host cell? If so, very few researchers have technologies in their pipelines that can meet these requirements. Should a first dose of phages be given and then treatment adjusted on the basis of phage resistant mutants test results? How do the parameters (e.g.; speed, robustness of phages, cost, and user friendliness) fit with the different treatment settings? There is a need to develop phage technology that is able to provide rapid, exceptional viruses that produce less phage-resistant bacteria mutants in the target pathogens.

**Fig. 2:**
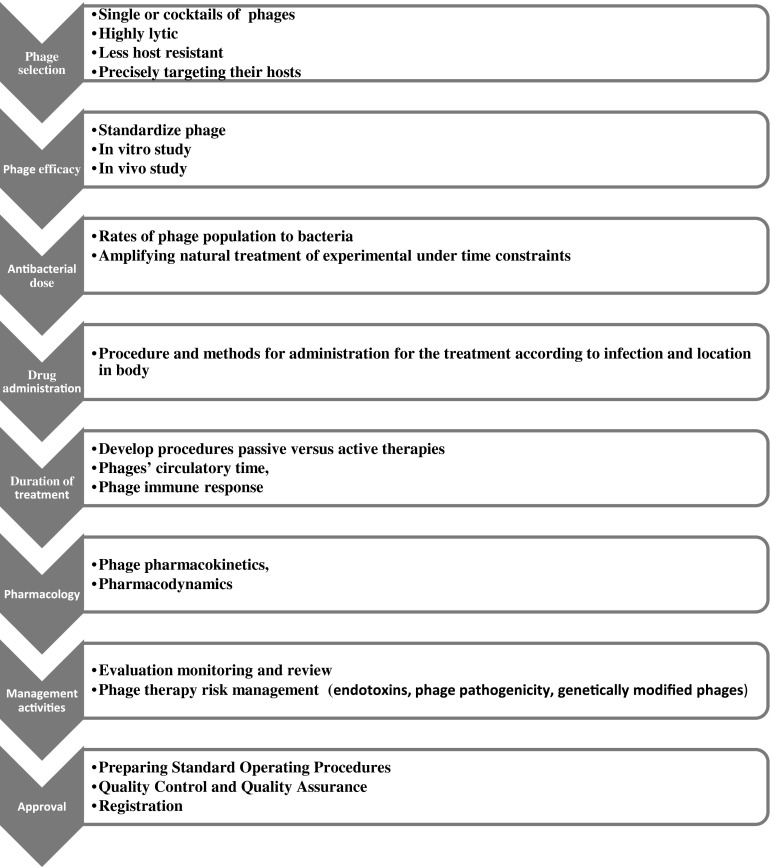
Development of a phage therapy management program

Figure 2 illustrates the main strands of a possible strategy based on analysis of the literature and reports published in peer reviewed/scholarly journals. The aim is to develop an action plan for safe phage therapy and business management to promote the use of phage therapy in appropriate health care applications.

In parallel there is an equal need for rapid detection methods, also able to detect swiftly any phage-resistant mutants so that corrective therapeutic measures can be taken before putting the patient’s life in danger. The identification of which organisms caused the infection using rapid detection methods is paramount, allowing doctors to know which phages are needed. Knowing which phage-resistant mutants are always expressed in vivo would allow these to also be targeted in the system. Many phage companies are struggling to align their business goals with the technology solutions because these fundamental questions have not been properly addressed by experts in the specialty. The world needs to rethink phage technology and realize that human and animal healthcare with the sharp increase of multi drug resistant bacteria, can be an economic driver that utilizes innovation fostered in the life science sector. Antibiotics are currently being phased out of animal production in many countries. Researchers are keen to continue to explore the science behind phage therapy uses, however, it remains unclear if phage therapy will indeed save lives on a significant scale or if it will ultimately fail to fulfil its promise. One thing seems clear though, if phage therapy is to move out of the twentieth century and into the twenty-first, so too must the regulatory models that govern it (Keen 2012). Obviously classical phage therapy did not produce consistently favourable results leaving antibiotics the preferred treatment. Though many believe that phages will not replace antibiotics right away or maybe ever, there is definite potential for their use in conjunction with antibiotics (Clark and March 2006).

## Conclusion

Phages are presenting solutions that will help to replace, curb, or promote judicious use of antibiotics in farm animals. Phage therapeutic approaches are also appropriate as adjunctive therapies to increase the efficacy of antibiotic treatment while simultaneously supporting probiotic supplements and useful microflora. As yet phage clinical therapy is in a progressive and scientific cumulative stage. To move into the pharmaceutical stage, it needs competencies, best practices and data that can support pharmaceutical industries to develop phage therapy ‘live drugs’. Innovative phage treatments will benefit from a new understanding of the phage-host–pathogen and environmental interactions. These are long-term solutions to the challenge of antibiotic resistance, driven by the urgent and growing need for new treatments.

## Abbreviations

CDC: Centres for Disease Control and Prevention
EFSA: European Food Safety Authority
FAO: Food and Agriculture Organization of the United Nations
FDA: Food and Drug Administration
FSA: Food Standards Agency
GPAs: Growth promoting antibiotics
GRAS: Generally recognized as safe
LPS: Lipopolysaccharide
MR: Multiplicity reactivation
OIE: World Organization for Animal Health
R&D: Research and Development
US-FSIS: US-Food Safety and Inspection Service
WHO: World Health Organization
